# A Novel Allosteric Activator of Free Fatty Acid 2 Receptor Displays Unique G_i_-functional Bias*

**DOI:** 10.1074/jbc.M116.736157

**Published:** 2016-07-05

**Authors:** Daniele Bolognini, Catherine E. Moss, Karolina Nilsson, Annika U. Petersson, Iona Donnelly, Eugenia Sergeev, Gabriele M. König, Evi Kostenis, Mariola Kurowska-Stolarska, Ashley Miller, Niek Dekker, Andrew B. Tobin, Graeme Milligan

**Affiliations:** From the ‡ Centre for Translational Pharmacology, Institute of Molecular, Cell and Systems Biology, College of Medical, Veterinary and Life Sciences, University of Glasgow, Glasgow G12 8QQ, Scotland, United Kingdom,; the § Medical Research Council Toxicology Unit, University of Leicester, Leicester LE1 9HN, United Kingdom,; the ¶ Cardiovascular and Metabolic Diseases,; ‖ Respiratory, Inflammatory and Autoimmune Diseases Innovative Medicines and Early Development Biotech Unit, Department of Medicinal Chemistry, and; the ‖‖ Discovery Sciences, Reagents and Assay Development, AstraZeneca, Mölndal, Pepparedsleden 1, SE-431 83, Mölndal, Sweden,; the ** Institute of Cardiovascular and Medical Sciences, College of Medical, Veterinary and Life Sciences, University of Glasgow, Glasgow G12 8QQ, Scotland, United Kingdom,; the §§ Molecular, Cellular and Pharmacobiology Section,; ‡‡ Institute of Pharmaceutical Biology, University of Bonn, 53115 Bonn, Germany, and; the ¶¶ Institute of Infection, Immunity and Inflammation, College of Medical, Veterinary and Life Sciences, University of Glasgow, Glasgow G12 8QQ, Scotland, United Kingdom

**Keywords:** allosteric regulation, fatty acid, G protein, G protein-coupled receptor (GPCR), inflammation

## Abstract

The short chain fatty acid receptor FFA2 is able to stimulate signaling via both G_i_- and G_q_/G_11_-promoted pathways. These pathways are believed to control distinct physiological end points but FFA2 receptor ligands appropriate to test this hypothesis have been lacking. Herein, we characterize AZ1729, a novel FFA2 regulator that acts as a direct allosteric agonist and as a positive allosteric modulator, increasing the activity of the endogenously produced short chain fatty acid propionate in G_i_-mediated pathways, but not at those transduced by G_q_/G_11_. Using AZ1729 in combination with direct inhibitors of G_i_ and G_q_/G_11_ family G proteins demonstrated that although both arms contribute to propionate-mediated regulation of phospho-ERK1/2 MAP kinase signaling in FFA2-expressing 293 cells, the G_q_/G_11_-mediated pathway is predominant. We extend these studies by employing AZ1729 to dissect physiological FFA2 signaling pathways. The capacity of AZ1729 to act at FFA2 receptors to inhibit β-adrenoreceptor agonist-promoted lipolysis in primary mouse adipocytes and to promote chemotaxis of isolated human neutrophils confirmed these as FFA2 processes mediated by G_i_ signaling, whereas, in concert with blockade by the G_q_/G_11_ inhibitor FR900359, the inability of AZ1729 to mimic or regulate propionate-mediated release of GLP-1 from mouse colonic preparations defined this physiological response as an end point transduced via activation of G_q_/G_11_.

## Introduction

Short chain fatty acids (SCFAs)^2^ are carboxylic acids containing 1 to 6 carbons. These are produced in the body predominantly by the fermentation of non-digestible carbohydrates (fibers) through the metabolic activity of the gut microbiota, with acetate (C2), propionate (C3), and butyrate (C4) being the most abundant products (1, 2). Until recently, the role of SCFAs was believed to be confined to providing either a source of energy or as intermediate products of more complex molecules (1). For example, colonocytes utilize C4 as a main energy supply, whereas the liver can source C2 as a substrate for the synthesis of cholesterol or longer chain fatty acids and C3 as a precursor for gluconeogenesis (2). However, with the discovery and de-orphanization of a pair of G protein-coupled receptors (GPCRs) responsive to SCFAs, FFA2, and FFA3 (previously designated GPR43 and GPR41, respectively) (3), it became clear that the SCFAs also act as signaling molecules (4–6). The SCFA receptors are mainly expressed in tissues involved in metabolic regulation, in particular white adipose tissue, intestinal enteroendocrine cells, and pancreatic beta cells (7) and in immune cells (8). Hence, a role for the SCFA receptors, in particular FFA2, in controlling energy homeostasis, and potentially in the regulation of metabolic disorders, has been proposed (9–11).

Despite the increasing interest surrounding SCFA receptors, a general paucity of selective ligands has rendered the study and understanding of their pathophysiological functions extremely challenging (7, 12). For example, although there is a rank order of selectivity of endogenous SCFAs for the receptors (13), with C2 being more potent at human FFA2 than at FFA3, this level of selectivity, in concert with the general low potency of the SCFAs, is insufficient to allow their use in defining physiological roles of FFA2 over FFA3 without additional studies that incorporate receptor knock-down or knock-out models (7). Moreover, even this poor degree of SCFA ligand selectivity is absent in the mouse orthologs of the SCFA receptors, where C2 is equipotent in activating FFA2 and FFA3 (7, 14). To date only a small number of selective synthetic ligands have been identified, with “compound 1” (3-benzyl-4-(cyclopropyl-(4-(2,5-dichlorophenyl)thiazol-2-yl)amino)-4-oxobutanoic acid) and 4-CMTB (4-chloro-α-(1-methylethyl)-*N*-2-thiazolyl-benzeneacetamide) being the best characterized ligands with agonist activity at FFA2 (7, 12). Although compound 1 is a selective and relatively potent orthosteric agonist at both human and mouse FFA2 (15), other representatives from this chemical series display markedly lower potency at murine FFA2 (15), limiting their potential for studies in murine cells and tissues (7). Equally, although 4-CMTB acts as both a direct allosteric agonist and also as a positive allosteric modulator (PAM) of the action of SCFAs at human FFA2 (16–19), and displays similar activity at mouse FFA2, the poor pharmacokinetic properties of this ligand (17) have limited its use within *in vivo* validation studies for FFA2.

An interesting feature of FFA2 is that, unlike the closely related SCFA receptor FFA3, which signals only via G_i_-family G proteins, it is able to initiate signals via both G_i_ and G_q_/G_11_-mediated pathways (7, 13). However, the contribution of these two signaling arms to various downstream cascades and, indeed, to physiological processes remains, in large part, undefined. Clearly, mechanistically distinct and potent classes of synthetic ligands are required to define such questions. To begin to address this need, herein, we characterize a novel synthetic ligand *N*-[3-(2-carbamimidamido-4-methyl-1,3-thiazol-5-yl)phenyl]-4-fluorobenzamide (AZ1729). We demonstrate that AZ1729 acts as a FFA2 allosteric agonist and PAM by selectively activating G_i_ signaling but lacks the capacity to activate FFA2 G_q_/G_11_-mediated signaling pathways. In this way we define AZ1729 as a G_i_-biased ligand. In conjunction with the use of selective inhibitors of the G_i_ and G_q_/G_11_ classes of G proteins, we then employ AZ1729 to define the relative contribution of these pathways to the integration of ERK1/2-MAP kinase activity in transfected cells and in natively expressing systems, in which we show that both FFA2-mediated inhibition of lipolysis and promotion of neutrophil chemotaxis reflect signaling via G_i_ family G proteins. By contrast, in murine colonic crypts FFA2 signaling to promote release of glucagon-like peptide-1 (GLP-1) is not modulated by AZ1729 indicating that this response proceeds via activation of G_q_/G_11_.

## Results

### 

#### 

##### AZ1729 Activates FFA2-dependent G_i_ Signaling

**FIGURE 1.**
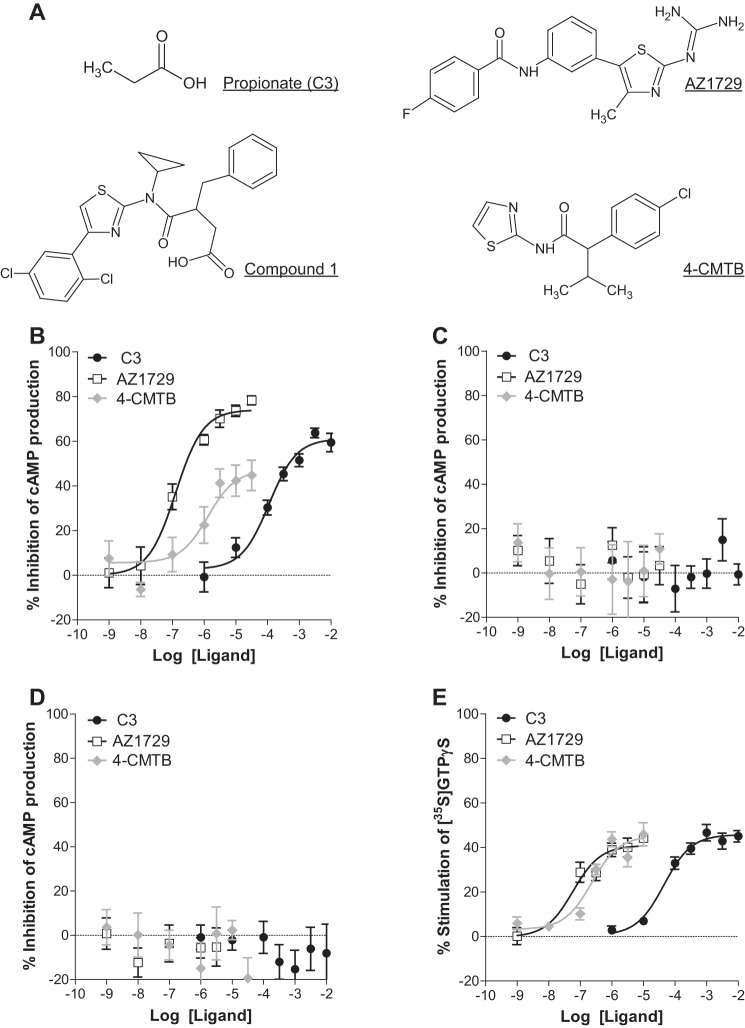
**AZ1729 is a potent activator of G_i_-dependent FFA2 signaling.** Chemical structures of C3, AZ1729, compound 1, and 4-CMTB are shown (*A*). The effect of C3, 4-CMTB, and AZ1729 on forskolin-induced stimulation of cAMP production in Flp-In^TM^ T-REx^TM^ 293 cells that had been induced to express hFFA2 (*B*) or such cells that were pre-treated with PTX (*D*) is shown. Compound effects require hFFA2 as without induction of the receptor no responses were observed (*C*). The effect of C3, 4-CMTB, and AZ1729 on [^35^S]GTPγS incorporation in membranes of hFFA2 expressing cells (*E*). Data are expressed as percentage inhibition of forskolin-stimulated cAMP production (*B–D*) or percent stimulation of [^35^S]GTPγS binding (*E*). Results represent mean ± S.E. (*n* = 9–12).

In Flp-In^TM^T-REx^TM^ 293 cells (20) able to express human (h)FFA2-eYFP only upon addition of the antibiotic doxycycline, the physiological FFA2 receptor agonist propionate (C3) (Fig. 1*A*) inhibited forskolin-stimulated cAMP levels in a concentration-dependent manner (pEC_50_ = 3.95 ± 0.13) (Fig. 1*B*). AZ1729 (Fig. 1*A*), which was identified initially as a regulator of FFA2 in a high throughput screen conducted using an integrative, function agnostic, dynamic mass redistribution assay, also inhibited forskolin-stimulated cAMP levels in these cells and, equally, did so in a concentration-dependent manner (pEC_50_ = 6.90 ± 0.14) (Fig. 1*B*). AZ1729 was somewhat more efficacious in this assay than C3 (74.0 ± 3.1% and 61.1 ± 2.8% inhibition of forskolin-stimulated cAMP levels, respectively) and also more than 500-fold more potent (*p* < 0.01). By contrast, a previously described allosteric agonist of this receptor 4-CMTB (16, 18) (Fig. 1*A*), although also able to inhibit forskolin-stimulated cAMP levels (pEC_50_ = 5.88 ± 0.39) was clearly a partial agonist (Fig. 1*B*). These effects were dependent on the presence of hFFA2 as none of C3, AZ1729, and 4-CMTB affected cAMP levels in these cells when expression of the hFFA2 receptor was not induced (Fig. 1*C*). As anticipated, this effect of each ligand was transduced via pertussis toxin (PTX)-sensitive G_i_-family G proteins. Treatment of doxycycline-induced cells with PTX, to cause ADP-ribosylation of the α subunit of members of this G protein subgroup, eliminated ligand regulation of cAMP levels in each case (Fig. 1*D*). The ability of AZ1729 to induce G_i_-mediated FFA2 signaling was also assessed in [^35^S]GTPγS binding assays, an end point that is best suited to, and usually reflects, the contribution of G_i_ family G proteins (21). In membranes prepared from Flp-In^TM^ T-REx^TM^ 293 cells induced to express hFFA2-eYFP, AZ1729 was able to produce stimulation of [^35^S]GTPγS binding with pEC_50_ 7.23 ± 0.20 (Fig. 1*E*). Again, this compound displayed potency that was significantly higher than both C3 (pEC_50_ 4.35 ± 0.14, *p* < 0.01) and 4-CMTB (pEC_50_ 6.50 ± 0.16, *p* < 0.05), although in this case without marked differences in ligand efficacy (Fig. 1*E*).

##### AZ1729 Displays G_i_-functional Bias at FFA2

**FIGURE 2.**
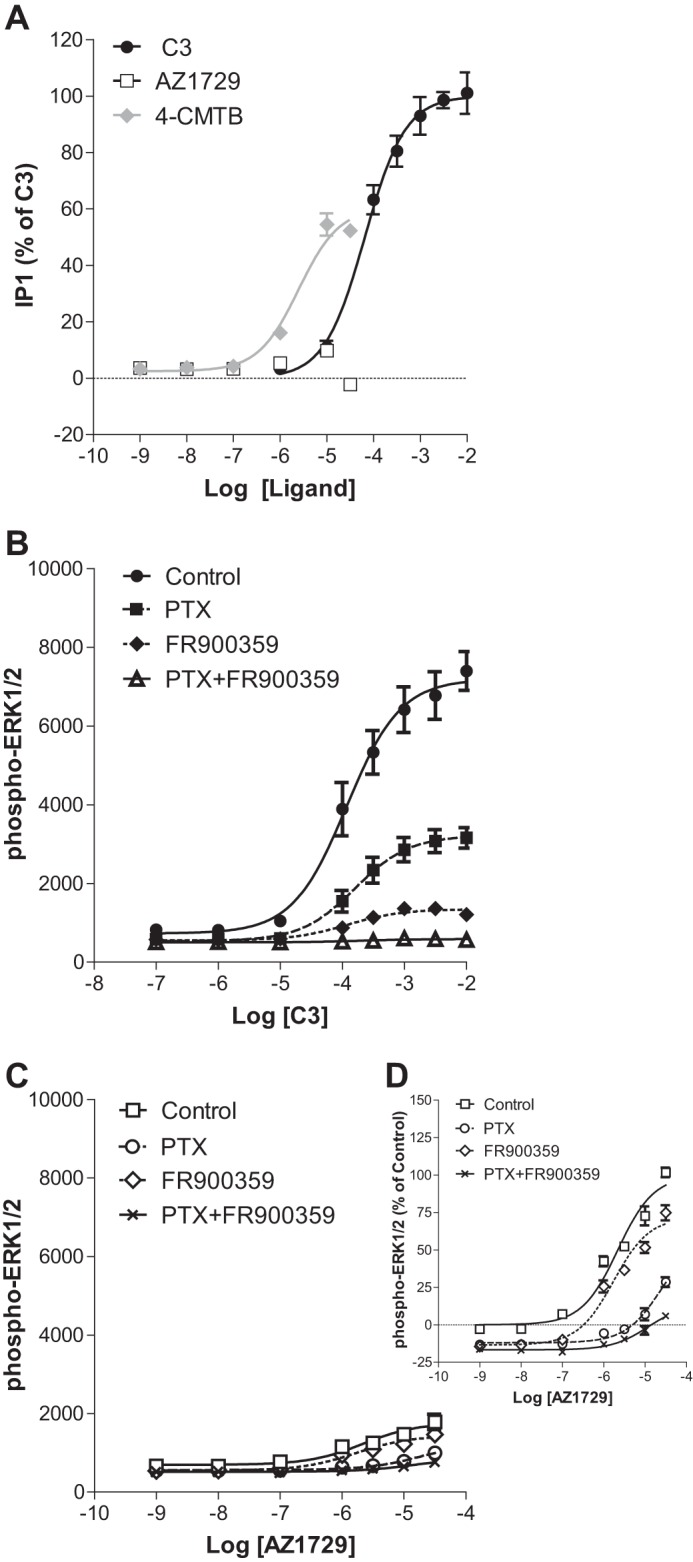
**AZ1729 is a G_i_-biased regulator of FFA2.** The effect of C3, 4-CMTB, and AZ1729 on IP_1_ accumulation assay in Flp-In^TM^ T-REx^TM^ 293 cells, induced to express hFFA2, is shown (*A*). The effect of C3 (*B*) and AZ1729 (*C*) on ERK1/2 phosphorylation in Flp-In^TM^ T-REx^TM^ 293 cells induced to express hFFA2 that had been pre-treated or not with PTX and/or FR900359. Data are expressed as the percentage of IP_1_ accumulation taking as 100% the maximal effect of C3 (*A*), or ERK1/2 phosphorylation signal ratio between 665 and 620 nm multiplied by a factor of 10,000 (*B* and *C*). *D,* displays the same data as *C* but presents the information % of the maximal effect of AZ1729 in untreated FFA2 expressing cells (control). Results represent mean ± S.E. (*n* = 6–8).

As well as transducing signals via “G_i_” the FFA2 receptor is appreciated to be able to also interact with, and signal via, G_q_/_11_ G proteins (4–6). Indeed, in cells induced to express hFFA2-eYFP, C3 produced a large increase in inositol monophosphate (IP_1_) accumulation (Fig. 2*A*), with potency (pEC_50_ = 4.19 ± 0.10) akin to that observed in the cAMP regulation experiments. Although 4-CMTB also produced a partial effect in this assay (pEC_50_ = 5.60 ± 0.11), AZ1729 was completely inactive at concentrations up to the highest (30 μm) that could be tested (Fig. 2*A*). These observations indicated that AZ1729 might be a “G_i_-biased” agonist at hFFA2. To assess this hypothesis we also explored ligand regulation of phospho-ERK1/2, production of which can be induced by both G_i_- and G_q/11_-mediated signaling. C3 produced a robust increase of phospho-ERK1/2 levels (Fig. 2*B*) with potency (pEC_50_ = 3.93 ± 0.13) similar to those displayed in both G_i_- and G_q/11_-dependent assays. Interestingly, the effect of C3 on the phosphorylation of ERK1/2 was partially inhibited by pre-treatment of the cells with PTX and greatly reduced by the G_q_/G_11_ inhibitor FR900359 (22) (Fig. 2*B*), suggesting that although C3 mediates phospho-ERK1/2 accumulation via both G protein classes it is mainly through G_q_/G_11_. In the same assay, AZ1729 was only a very weak partial agonist at this end point (Fig. 2, *C* and *D*) and also displayed more modest potency (pEC_50_ = 5.66 ± 0.21). Interestingly, the effect of AZ1729 on phospho-ERK1/2 accumulation was completely abolished in hFFA2 cells pre-treated with PTX, but unaffected by treatment with FR900359 (Fig. 2, *C* and *D*). The effects of both C3 and AZ1729 were completely abrogated upon pre-treatment of cells with both PTX and FR900359 (Fig. 2, *B–D*). Together, these data indicate that AZ1729 behaves as a biased agonist at hFFA2; preferentially activating G_i_ but being unable to engage with G_q/_G_11_ signaling.

##### AZ1729 Does Not Interact with the Orthosteric Binding Site of FFA2

**FIGURE 3.**
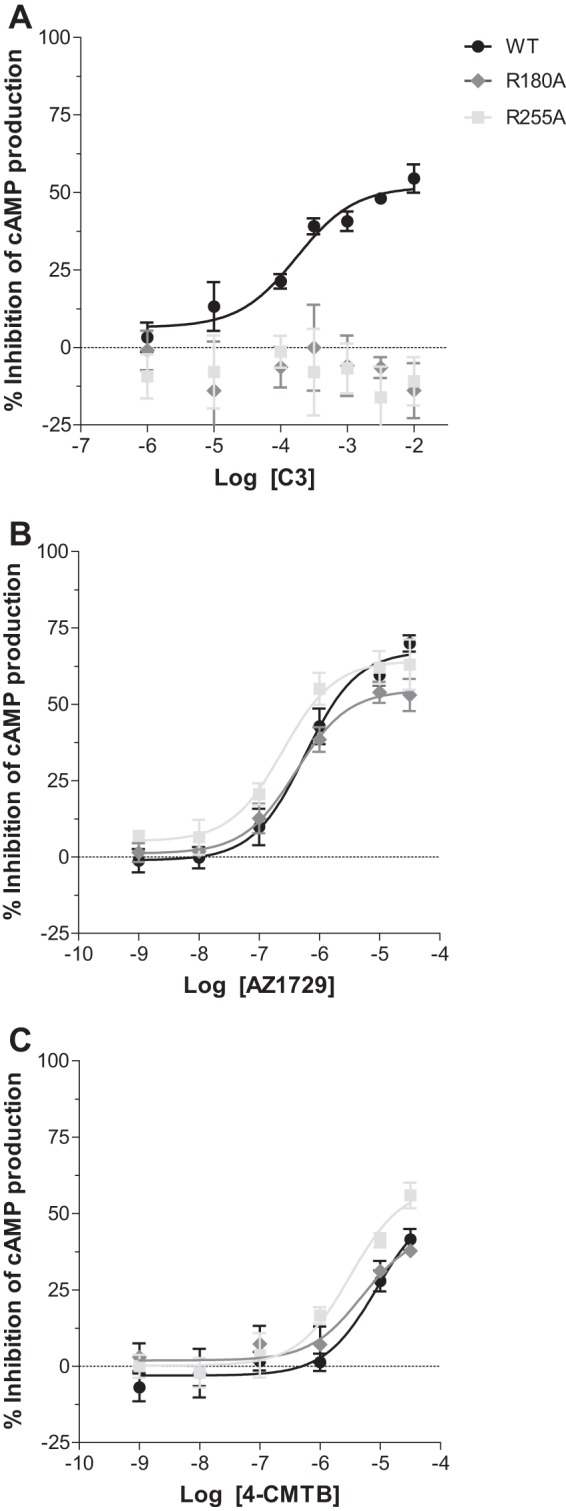
**AZ1729 signaling via FFA2 is not affected by mutations in the orthosteric binding pocket.** The effect of C3 (*A*), AZ1729 (*B*), and 4-CMTB (*C*) on forskolin-induced stimulation of cAMP production in Flp-In^TM^ T-REx^TM^ 293 cells that had been induced to express wild type, R180A or R255A mutants for hFFA2. Data are expressed as the percentage inhibition of cAMP production. Results represent the mean ± S.E. (*n* = 6).

**FIGURE 4.**
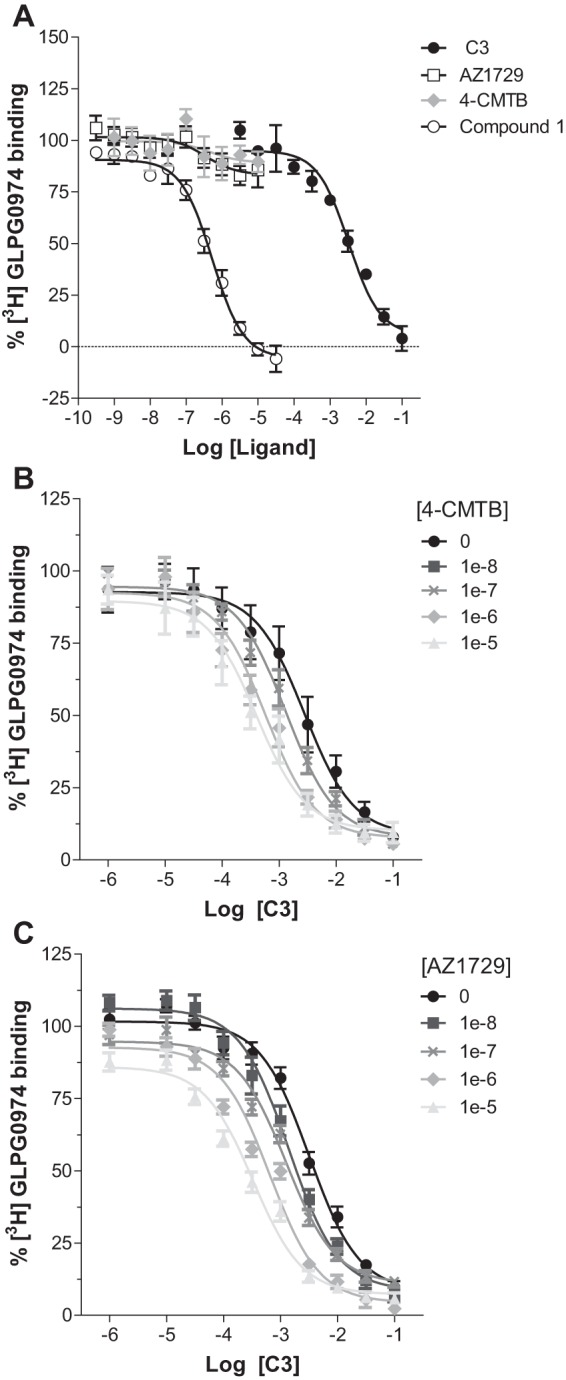
**AZ1729 is a positive allosteric regulator of C3 affinity at FFA2.** The ability of varying concentrations of C3, AZ1729, 4-CMTB, and compound 1 to compete with [^3^H]GLPG0974 in equilibrium competition binding experiments in membranes of Flp-In^TM^ T-REx^TM^ 293 cells induced to express hFFA2 is shown (*A*). The effect of increasing concentrations of 4-CMTB (*B*) or AZ1729 (*C*) on the ability of C3 to compete with [^3^H]GLPG0974 binding in membranes expressing hFFA2 is displayed. Data are expressed as the percentage of specific [^3^H]GLPG0974 binding. Results represent the mean ± S.E. (*n* = 6–10).

Synthetic agonists at FFA2 that share the same orthosteric binding site as C3, and the other endogenously produced SCFAs, all contain a carboxylate group and this is integral to their agonist function (14, 15, 23, 24). AZ1729 does not, and neither does it contain a bioisostere that might substitute for the carboxylate (Fig. 1*A*). We examined, therefore, if AZ1729 acted as an orthosteric agonist. This was assessed in two distinct ways. First, we examined whether the G_i_-mediated agonism of AZ1729 was preserved in orthosteric binding site mutants of hFFA2. Two arginine residues, Arg-180 and Arg-255 (Ballesteros and Weinstein residue location numbers 5.39 and 7.35, respectively) act to coordinate the carboxylate moiety of FFA2 orthosteric agonists (23). Mutation of either of these residues to Ala was able to eliminate the ability of C3 to inhibit forskolin-stimulated cAMP production (Fig. 3*A*) but did not affect substantially the function of AZ1729 (Fig. 3*B*). Moreover, as described previously (18), this was also the case for the allosteric agonist 4-CMTB (Fig. 3*C*). These data indicate that neither AZ1729 nor 4-CMTB interact with the arginine residues that are the core of the FFA2 orthosteric binding pocket (23) and are, therefore, allosteric agonists. Second, we performed sets of radioligand binding experiments. [^3^H]GLPG0974 was recently reported as an orthosteric radiolabeled antagonist of hFFA2 (25). In membranes from Flp-In^TM^ T-REx^TM^ 293 cells induced to express hFFA2, [^3^H]GLPG0974 bound with high affinity (*K_d_* = 7.5 nm) (25) and this was fully outcompeted by increasing concentrations of C3 (p*K_i_* = 2.78 ± 0.11) (Fig. 4*A*). This was also the case when using the previously described hFFA2 receptor synthetic orthosteric agonist compound 1 (15) (Fig. 1*A*) (p*K_i_* = 6.58 ± 0.09) (Fig. 4*A*). However, AZ1729 was unable to compete fully with [^3^H]GLPG0974 for binding to the receptor (Fig. 4*A*), demonstrating that AZ1729 does not share an overlapping binding site with the radiolabeled antagonist. It did, however, produce maximally 16.7 ± 4.4% reduction in specific binding of this radioligand with apparent p*K_i_* = 6.77 ± 0.50 (Fig. 4*A*). 4-CMTB was also poor in capacity to compete with [^3^H]GLPG0974, maximally preventing only 10.9 ± 6.8% of the specific binding of this ligand with apparent p*K_i_* estimated as 6.64 ± 1.29 (Fig. 4*A*).

##### AZ1729 Interacts with a FFA2 Allosteric Binding Site

The unaltered functional effect of AZ1729 at orthosteric binding site mutants of hFFA2, and also the inability of this compound to fully displace [^3^H]GLPG0974, raised the possibility that AZ1729 might interact with an allosteric binding site on hFFA2. To test this hypothesis we performed a three-way radioligand binding equilibrium experiment (26). In particular, we assessed the ability of 4-CMTB and AZ1729 to modulate the inhibition of [^3^H]GLPG0974 binding by the endogenous orthosteric agonist, C3. Increasing concentrations of 4-CMTB produced a leftward shift of the C3 concentration-response curve, indicating that 4-CMTB increases the affinity of C3 to inhibit [^3^H]GLPG0974 binding (Fig. 4*B*). Similar results were obtained with AZ1729, although this compound, in addition to a leftward shift of the C3 concentration-response curve, produced a downward shift (Fig. 4*C*) as anticipated from the modest but detectable capacity of AZ1729 to directly modulate binding of [^3^H]GLPG0974. Using an extended allosteric ternary complex model equation (see “Materials and Methods”), we quantified the affinity cooperativity factor of 4-CMTB and AZ1729 *versus* [^3^H]GLPG0974 (α) and C3 (α′). This showed that 4-CMTB did not have a significant cooperativity effect toward [^3^H]GLPG0974 binding (α = 0.93, *i.e.* close to unity), whereas AZ1729 displayed a weak negative cooperativity effect (α = 0.67). In contrast, both 4-CMTB and AZ1729 displayed positive cooperativity for C3 binding (α′ = 4.45 ± 1.16 and 4.27 ± 1.17, respectively). From the same equation it was possible to calculate the affinity of these synthetic compounds for hFFA2 (p*K_B_* = 6.52 ± 0.17 for 4-CMTB and 6.84 ± 0.11 for AZ1729). Together, these data indicate that AZ1729, as for 4-CMTB, binds to a FFA2 allosteric binding site.

##### AZ1729 Is a Functional Positive Allosteric Modulator in FFA2-mediated G_i_ Signaling

**FIGURE 5.**
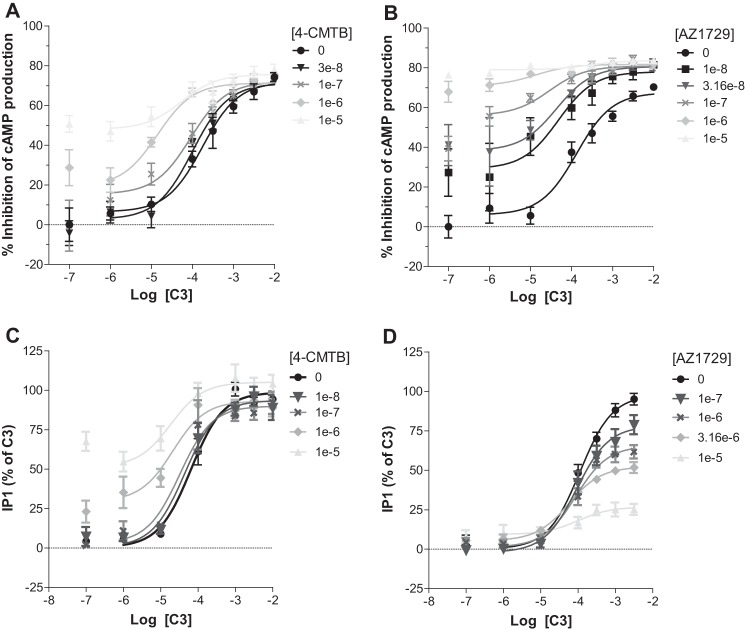
**AZ1729 is a positive allosteric modulator of hFFA2-mediated G_i_ signaling.** The effect of increasing concentrations of 4-CMTB (*A* and *C*) or AZ1729 (*B* and *D*) on C3 regulation of forkolin-induced cAMP production (*A* and *B*) or IP_1_ accumulation (*C* and *D*). Data are expressed as the percentage inhibition of cAMP production (*A* and *B*) or IP_1_ accumulation taking as 100% the maximal response of C3 (*C* and *D*). Results represent the mean ± S.E. (*n* = 6–10).

Having established that AZ1729 displays positive cooperativity toward the binding of C3 to hFFA2, we investigated whether this property would also be observed in functional assays. 4-CMTB has been reported to act as both a direct agonist of the FFA2 receptor and also as an effective PAM of the activity of C3 (16, 18). This was confirmed in cAMP assays in cells induced to express hFFA2 where, as well as acting directly as a partial agonist, 4-CMTB also increased, in a concentration-dependent fashion, the observed potency of C3 (Fig. 5*A*). Application of allosteric parameter fitting models allowed estimation of the affinity (p*K_B_*) of 4-CMTB as 5.68 ± 0.17 and with the ligand displaying strong positive net cooperativity (αβ = 28.35). In the same assay AZ1729 displayed very strong positive net cooperativity *versus* C3, with αβ = 85.22. Because AZ1729 effects on affinity of C3 were modest (*i.e.* α′ = 4.27 ± 1.17) the functional cooperativity of AZ1729 observed here in the C3 cAMP response can be largely attributed to enhanced efficacy of C3 in the presence of AZ1729. Estimated affinity of AZ1729 for the receptor was 6.75 ± 0.12 (Fig. 5*B*), similar to the value obtained from the radioligand binding experiments.

These experiments also demonstrated that in addition to AZ1729 showing positive functional cooperativity in the C3 cAMP response this ligand also showed intrinsic agonist activity, confirming the data in Fig. 1*B* that AZ1729 acts as a full agonist (Fig. 5*B*). Because we are able to estimate the affinity of AZ1729 and 4-CMTB from the binding experiments (Fig. 4*A*) the agonist activity of both of these allosteric modulators can be analyzed by fitting the cAMP concentration-response curves (Figs. 1*B* and 5*B*) to the operational model to generate a measure of the transduction efficacy (τ). Using this approach AZ1729 had a τ = 5.77 ± 1.22 compared with a τ value of 1.62 ± 1.22 for 4-CMTB, indicating that AZ1729 drives stronger coupling of FFA2 to the cAMP signaling pathway than the PAM-agonist 4-CMTB.

##### AZ1729 Is a Functional Negative Allosteric Modulator in hFFA2-mediated G_q_/G_11_ Signaling

Although lacking direct agonism, we next assessed whether AZ1729 also displayed positive cooperativity in the G_q_/G_11_-dependent IP_1_ accumulation assay. First, we assayed 4-CMTB, which has already been reported as a PAM in other G_q_/G_11_-dependent assays (18). Herein 4-CMTB acted as an agonistic PAM of C3 with αβ = 13.88 and estimated affinity (p*K_B_*) = 5.47 ± 0.20 (Fig. 5*C*). Surprisingly, in the same assay AZ1729 acted instead as an insurmountable/allosteric antagonist, as it was able to reduce C3 maximal efficacy in a concentration-dependent manner, with no significant changes in C3 affinity (Fig. 5*D*). Estimated affinity for AZ1729 in this assay was 6.12 ± 0.12 (Fig. 5*D*).

**FIGURE 6.**
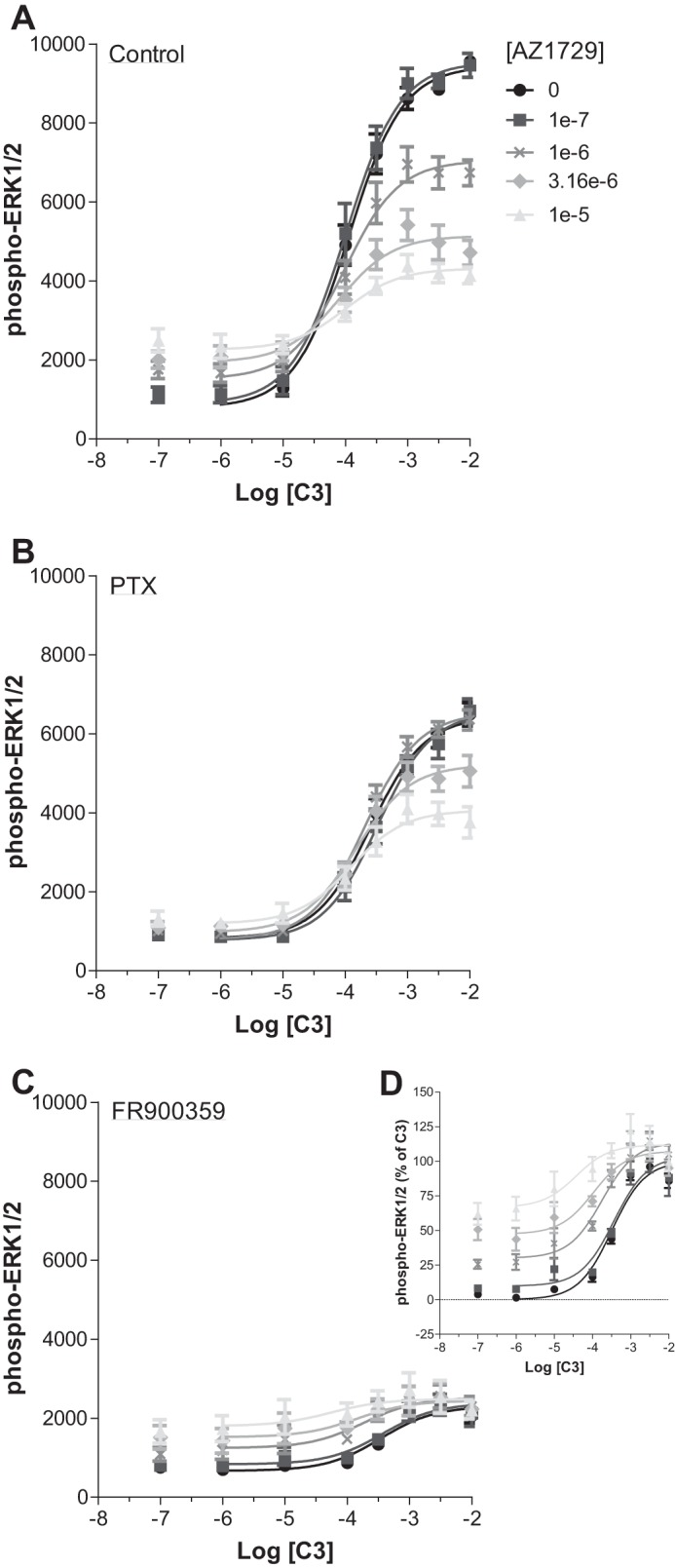
**AZ1729 displays differential FFA2-mediated G_i_ and G_q/11_ signaling effects: studies on ERK1/2 phosphorylation.** The effect of increasing concentrations of AZ1729 on C3 concentration-response curves on ERK1/2 phosphorylation assays in hFFA2 expressing cells that were untreated (*A*) or had been pre-treated with PTX (*B*) or with FR900359 (*C*). *D,* displays the same data as *C* but presents the information as % of the maximal effect of C3 in FR900359 pre-treated hFFA2 expressing cells. Results represent mean ± S.E. (*n* = 6).

The ability of AZ1729 to act as a PAM in G_i_-coupled FFA2 assays but as an insurmountable/allosteric antagonist in G_q_/G_11_-mediated FFA2 end points was further assessed in the phospho-ERK1/2 assay. As noted earlier (Fig. 2*B*), at FFA2, C3 causes accumulation of phospho-ERK1/2 mainly through activation of G_q_/G_11_. Increasing concentrations of AZ1729, as well as displaying weak partial agonism, were able to induce a negative effect on C3 maximal efficacy (Fig. 6*A*). To understand the contribution of FFA2-mediated G_i_ *versus* G_q_/G_11_ signaling, cells were pre-treated with PTX or FR900359. After pre-treatment with PTX to ablate G_i_-mediated signaling and allow detection only of G_q_/G_11_-mediated signals, AZ1729 retained its ability to induce a decrease in the maximal efficacy of the C3 concentration-response curve, but lost its own intrinsic partial agonism (Fig. 6*B*). By contrast, in FR900359-pretreated cells, and therefore examining only G_i_-mediated links to this end point, AZ1729 did not show any negative effect on the C3 concentration-response curve, but it rather behaved as an agonistic PAM, causing an increase in affinity of C3 and displaying intrinsic partial agonism (Fig. 6, *C* and *D*). Again these data confirm the functional bias effect of AZ1729 at hFFA2, behaving as an agonistic PAM in G_i_-mediated signaling but as a negative allosteric/insurmountable antagonist in G_q_/G_11_-mediated signaling.

##### AZ1729 Displays Probe Dependence at hFFA2

**FIGURE 7.**
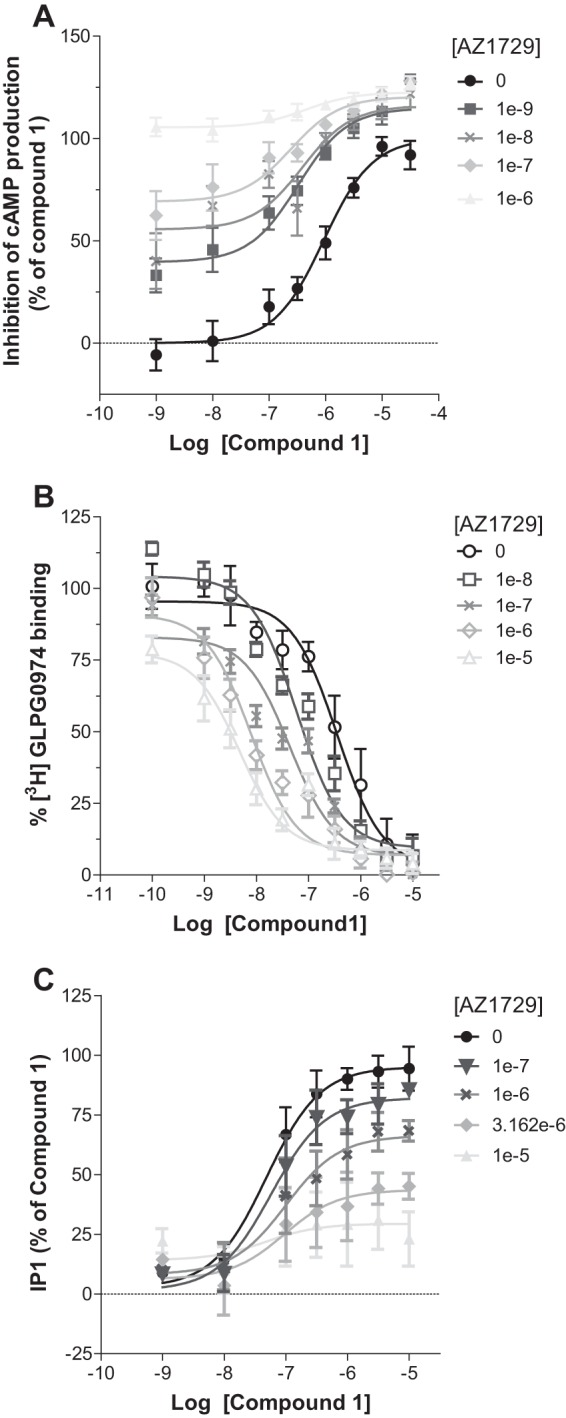
**AZ1729 is a potent allosteric modulator of compound 1 at hFFA2.** The effect of increasing concentrations of AZ1729 on compound 1 concentration-response curves in forskolin-induced cAMP production (*A*), competition binding studies using [^3^H]GLPG0974 (*B*), and IP_1_ accumulation (*C*) assays is shown. Results represent mean ± S.E. (*n* = 6–10).

A common feature of allosteric modulators is that they may display “probe dependence,” *i.e.* that the characteristics of the allosteric modulation can vary with the identity of the orthosteric agonist examined (27–29). Because compound 1, the most potent orthosteric FFA2 agonist yet described (15), is both substantially larger, and chemically distinct, from the endogenous agonists, we next assessed whether the differential effects of AZ1729 in pathways mediated by distinct G protein subtypes were preserved when using compound 1. To test this, increasing concentrations of AZ1729 were assayed together with compound 1 in both cAMP and IP_1_ assays. As with C3, AZ1729 was able to increase, in a concentration-dependent manner, the observed potency of compound 1 to regulate forskolin-induced cAMP accumulation (Fig. 7*A*). Application of allosteric parameter fitting models resulted in an apparent p*K_B_* value of 8.39 ± 0.11 and with the ligands displaying positive net cooperativity, αβ = 12.09. The high affinity value obtained in this experiment prompted us to assess whether AZ1729 would display a similar value also in a three-way radioligand binding experiment in the presence of compound 1. AZ1729 was able to strongly, and in a concentration-dependent manner, induce a leftward shift of the ability of compound 1 to compete with [^3^H]GLPG0974 for binding, with a maximum 100-fold increase in compound 1 affinity in the presence of 10 μm AZ1729 (Fig. 7*B*). Application of the allosteric ternary complex model equation resulted in α′ =47.29 ± 0.12 and p*K_B_* = 6.51 ± 0.13, defining that AZ1729 shows a higher cooperativity value toward compound 1 than toward C3. We next tested the effect of AZ1729 in the G_q_/G_11_-dependent IP_1_ assay. AZ1729 also caused a decrease in the maximal efficacy of compound 1 (Fig. 7*C*). Together, these results suggest that AZ1729, at least for compound 1, preserves its bias effect at FFA2. However, AZ1729 does display some probe dependence, having a higher cooperativity factor for compound 1 than for C3.

##### AZ1729 Displays FFA2 Species Ortholog-dependent Effects

**FIGURE 8.**
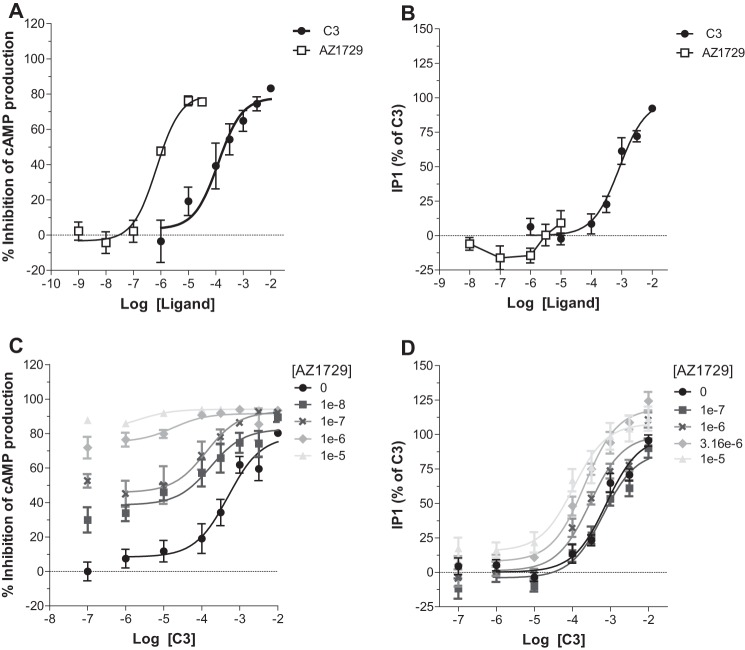
**AZ1729 also displays functional bias at mouse FFA2.** The effect of C3 or AZ1729 in Flp-In^TM^ T-REx^TM^ 293 cells induced to express mFFA2 on forskolin-induced cAMP production (*A*) or IP_1_ accumulation (*B*) assays is shown. The effect of increasing concentrations of AZ1729 on C3 concentration-response curves in forskolin-induced cAMP production (*C*) or IP_1_ accumulation (*D*) assays is also displayed. Results represent mean ± S.E. (*n* = 6–8).

Murine models often represent appropriate systems to test potential physiological effects of compounds. Mouse adipocytes reportedly express FFA2, and activation of this receptor has been demonstrated to inhibit lipolysis (30, 31). However, differences in ligand function between FFA2 species orthologs have been reported (14) and these might limit translational interpretation of outcomes in such models. As such AZ1729 was next assessed in both G_i_- and G_q_/G_11_-dependent assays in Flp-In^TM^ T-REx^TM^ 293 cells that were able to express mouse (m)FFA2-eYFP. In the cAMP assay, AZ1729 acted as an agonist with an efficacy similar to that displayed by C3 and with potency of 6.16 ± 0.12, significantly higher than C3 (*p* < 0.01) (Fig. 8*A*). Again, in the IP_1_ assay AZ1729 did not show any direct effect (Fig. 8*B*), confirming the bias behavior of this compound also at the mouse ortholog of FFA2.

We then moved to test whether the allosteric effects of AZ1729 were also retained at mFFA2. In cAMP accumulation experiments, co-addition of AZ1729 with C3 resulted in a concentration-dependent increase of C3 potency, suggesting that AZ1729 also acted as an agonistic PAM at mFFA2 (Fig. 8*C*). Application of the allosteric model equation resulted in an estimated p*K_B_* value of 7.17 ± 0.11 and a net cooperativity factor αβ = 13.49. In IP_1_ accumulation assays, AZ1729, although displaying no direct effect, was in this case able to induce a small increase in the potency of the C3 concentration-response curve (Fig. 8*D*) with a net cooperativity factor αβ = 20.01 and an estimated p*K_B_* value of 4.95 ± 0.29. Together these data suggest that, as for hFFA2, AZ1729 acts as allosteric agonist in G_i_-coupled end points. However, by contrast, the observed antagonism in G_q_/G_11_-coupled assays at hFFA2 was absent in mFFA2.

##### AZ1729 Induces Inhibition of Isoproterenol-induced Lipolysis in Mouse Adipocytes

**FIGURE 9.**
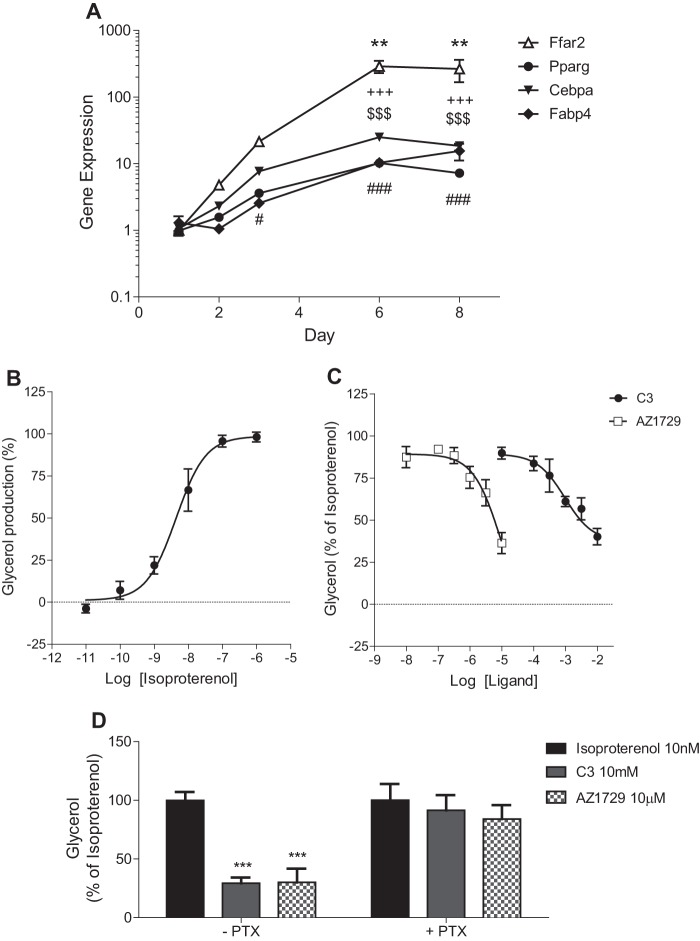
**AZ1729 inhibits lipolysis in primary mouse adipocytes.** Gene expression profiles of Ffar2, Pparγ, Cebpa, and Fabp4 in primary mouse adipocytes at days 1, 2, 3, 6, and 8 of differentiation (*A*). The effect of varying concentrations of isoproterenol on glycerol production in differentiated primary mouse adipocytes is displayed (*B*). The effect of increasing concentrations of C3 and AZ1729 on 10 nm isoproterenol-induced glycerol release in differentiated primary mouse adipocytes that were untreated (*C*) and/or had been pre-treated with PTX (*D*) is shown. Data are shown as expression of target genes relative to samples at day 1 of differentiation (*A*) or as percentage of glycerol release taking as 100% the maximal effect of 10 nm isoproterenol (*B–D*). Results represent mean ± S.E. (*n* = 4–8). **, *p* < 0.01; #, *p* < 0.05; ###, *p* < 0.001; +++, *p* < 0.001; $$$, *p* < 0.001 one-way analysis of variance followed by Dunnett's post hoc test (*A*). ***, *p* < 0.001 two-way analysis of variance followed by Bonferroni post hoc test (*D*).

Having established that AZ1729 is G_i_-biased we wanted to use this ligand to define the physiological signaling pathways employed by FFA2 in cells endogenously expressing FFA2. As previously stated, primary mouse adipocytes express FFA2, and activation of this receptor with SCFAs has been shown to inhibit lipolysis via a mechanism that is mainly G_i_-mediated (30, 31). Initially, we tested the expression levels of mFFA2 at different stages of differentiation in such primary cultures. Differentiation was monitored by measuring Pparγ, Cebpa, and Fabp4 mRNA expression levels (Fig. 9*A*). In parallel, mFFA2 mRNA levels increased with adipocyte differentiation, with significantly higher levels at days 6 and 8 of the differentiation protocol (Fig. 9*A*). By contrast, mRNA corresponding to the closely related SCFA receptor FFA3 was not detected in these primary murine adipocytes at any stage of differentiation (data not shown). We then tested the ability of the β-adrenoreceptor agonist, isoproterenol, to induce lipolysis in these cells, with the effect being measured by quantification of glycerol release. Isoproterenol induced a concentration-dependent release of glycerol, with pEC_50_ = 8.49 ± 0.18 (Fig. 9*B*), similar to that reported previously (32). C3 decreased isoproterenol-induced glycerol release (pEC_50_ = 3.07 ± 0.18) (Fig. 9*C*), also as reported previously (31). Similarly, AZ1729 was able to produce concentration-dependent (pEC_50_ = 5.03 ± 0.44) inhibition of the lipolytic effect of isoproterenol (Fig. 9*C*). To define whether these effects of both C3 and AZ1729 were G_i_-mediated, primary mouse adipocytes were pre-treated with PTX. The effects of both C3 and AZ1729 were completely abolished when G_i_ protein activity was blocked by PTX pre-treatment (Fig. 9*D*).

##### AZ1729 Induces Migration of Human Neutrophils

**FIGURE 10.**
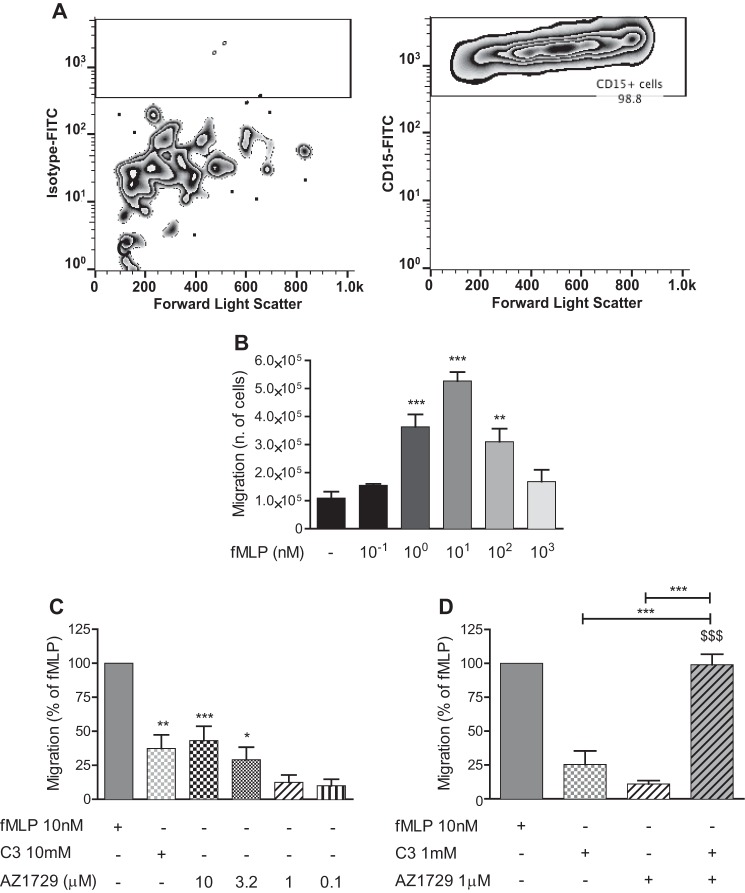
**AZ1729 induces human neutrophil migration.** Human neutrophils were labeled with isotype-FITC (*A*, *left panel*) or CD15-FITC (*A*, *right panel*) antibody and analyzed by FACS. The effect of increasing concentration of fMLP on human neutrophil migration (*B*). The effect of 10 nm fMLP, 10 mm C3, and increasing concentrations of AZ1729 on human neutrophil migration (*C*). The effect of single treatment or co-incubation of 1 mm C3 and 1 μm AZ1729 on human neutrophil migration is compared with the effect of 10 nm fMLP (*D*). Data are expressed as the number of migrated neutrophils (*B*) or as the percentage of migrated neutrophils taking as 100% the response to 10 nm fMLP (*C* and *D*). Results represent the mean ± S.E. (*n* = 4–7). *Forward light scatter* indicates cell size. Statistical analyses were performed by comparing the migratory response of each sample to vehicle-treated neutrophils (*B* and *C*). *, *p* < 0.05; **, *p* < 0.01; ***, *p* < 0.001 one-way ANOVA followed by Dunnett's post hoc test (*B* and *C*). ***, *p* < 0.001; $$$, *p* < 0.001 one-way analysis of variance followed by Bonferroni post hoc test (*D*).

The presence of FFA2 has been reported in cells of the innate immune system, where its expression is particularly marked in neutrophils (33–35). In these cells, FFA2 plays a role in chemotaxis, with a mechanism that seems to be PTX-sensitive, at least in mouse-derived neutrophils (36). We therefore assayed the effect of AZ1729 in human neutrophils. First we assessed that the isolated cells were positive for CD15, a marker for neutrophils. Results show that 98.8% of cells were indeed positive for this marker (Fig. 10*A*). Next we tested whether the isolated human neutrophils were able to migrate toward the well known chemoattractant, *N*-formyl-methionyl-leucyl-phenylalanine (fMLP). Results show that human neutrophils were able to migrate toward a concentration-gradient of fMLP (Fig. 10*B*), producing a standard “bell-shaped” response (37) with maximal migration induced by 10 nm fMLP. Both C3 and AZ1729 were also significantly able to induce neutrophil migration at 10 mm and 3–10 μm, respectively, although to a lesser extent than 10 nm fMLP (Fig. 10*C*). Because in the *in vitro* experiments AZ1729 behaved as an allosteric agonist in G_i_-dependent assays, we assessed the effect of AZ1729 on C3-mediated neutrophil migration. Neutrophil migration induced by 1 mm C3 significantly increased in the presence of 1 μm AZ1729, a concentration of the allosteric regulator that was unable to promote significant neutrophil migration when added alone (Fig. 10*D*), indicating that AZ1729 can potentiate the effect of C3 on neutrophil migration.

##### AZ1729 Does Not Regulate FFA2-mediated Release of GLP-1 from Mouse Colonic Crypts

**FIGURE 11.**
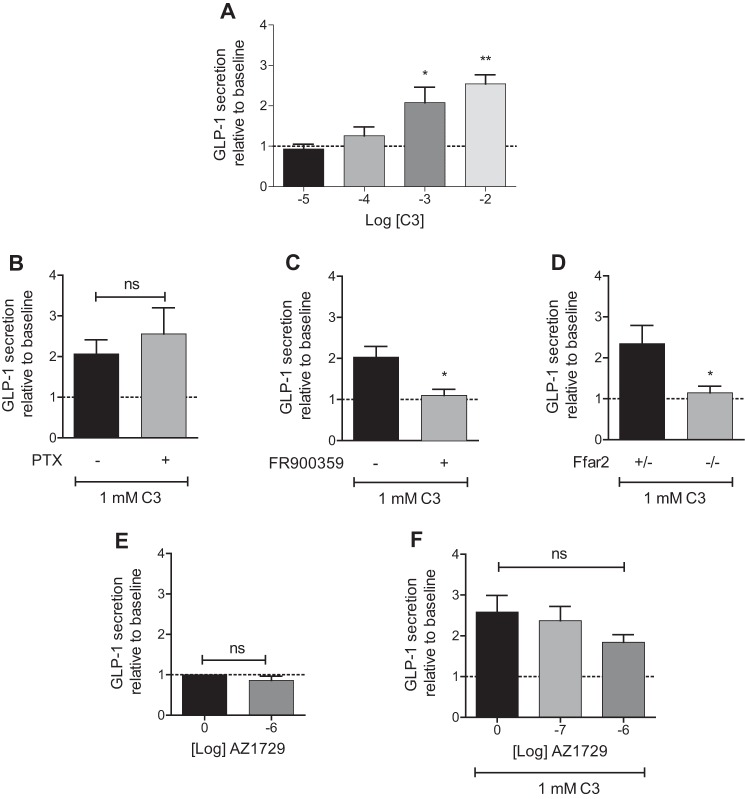
**AZ1729 does not regulate FFA2-mediated release of GLP-1 from mouse colonic crypts.** The effect of increasing concentrations of C3 on GLP-1 secretion in mouse colonic crypts (*A*). The effect of 1 mm C3 on GLP-1 secretion in mouse colonic crypts that had or had not been pretreated with PTX (*B*) or FR900359 (*C*). The effect of 1 mm C3 on GLP-1 secretion in mouse colonic crypts derived from heterozygous (+/−) or knock-out (−/−) mice for Ffar2 (*D*). The effect of AZ1729 alone (*E*) or in combination with 1 mm C3 (*F*) on GLP-1 secretion in mouse colonic crypts. *, *p* < 0.05; **, *p* < 0.01 one-way analysis of variance followed by Dunnett's post hoc test (*A* and *F*). *, *p* < 0.05 unpaired *t* test (*B–E*).

Production of colonic crypts from wild type mice allowed demonstration that C3 increased release of GLP-1, and did so in a concentration-dependent fashion (Fig. 11*A*). This ability of C3 was unaffected by pre-treatment with PTX but completely blocked by addition of FR900359 (Fig. 11, *B* and *C*), indicating this to be a G_q_/G_11_-mediated effect. As anticipated, C3 did not mediate GLP-1 secretion in preparations derived from Ffar2 knock-out animals (Fig. 11*D*). Consistent with this being a G_q_/G_11_-mediated pathway and the very limited effect of AZ1729 observed on G_q_/G_11_-mediated signaling in cells heterologously transfected to express mouse FFA2, at concentrations of 1 μm, AZ1729 did not affect GLP-1 release in mouse colonic crypts (Fig. 11*E*). Similarly, AZ1729 did not affect C3-mediated GLP-1 release (Fig. 11*F*).

## Discussion

Ligand bias, which is the ability of a compound to selectively promote transduction of information from a receptor via a subset of the panoply of signaling pathways the receptor can engage with, compared with the effects produced at the same pathways by a reference compound, is now well established and widely discussed (38–40). Initially, studies on ligand bias focused primarily on proof-of-concept and were frequently limited to experiments performed using receptors expressed in heterologous cell systems. However, experience derived from such studies has facilitated the translation of these ideas to native systems, to the extent that clinical trials on biased ligands for a number of receptors have been performed and more are ongoing (41–43). These are designed to deliver new medicines with high efficacy but with a significant reduction in side effects that reflect engagement with signaling pathways that are contraindicated in effective treatment. In many cases assessment of bias at GPCRs has focused largely on separation of the effectiveness of ligands to regulate signals that reflect either “canonical” G protein-dependent or “non-canonical,” non-G protein-dependent effects. The non-G protein-dependent effects are routinely assessed as interactions with arrestins, in part because a variety of assays allow easy measurement of such interactions but, also in part, because it is clear that arrestins allow both kinetically and spatially distinct signals to be developed after ligand engagement with a GPCR (44, 45). Despite this, it is clear that many GPCRs can interact with and signal via members of more than one of the four broad families of heterotrimeric G proteins. As such, ligand bias can also be generated between activation of such G protein families. In early days such studies centered on the ability of ligands to selectively activate G_s_ *versus* G_i_-mediated pathways but in recent times the development of a wide range of G protein subtype selective biosensors has resulted in more global assessments of ligand and receptor bias across the G protein family (46–49).

Initial deorphanization studies on FFA2 (then designated GPR43) highlighted the capacity of this receptor to regulate both G_i_ and G_q_/G_11_-mediated signals when stimulated with SCFAs (5) and subsequent work has confirmed this dual signaling capacity. Moreover, until now this has also been the case for synthetic activators of FFA2, whether binding to the orthosteric (15) or to an allosteric (16, 18–19) site.

AZ1729 was identified initially in a high throughput screen designed to discover regulators of FFA2. As this compound is devoid of a carboxylic acid moiety, which appears to be essential for orthosteric agonist actions at FFA2 (25), it was anticipated to act as an allosteric regulator. This was confirmed in that AZ1729 maintained agonist function and potency at orthosteric binding site mutants of FFA2, was unable to effectively outcompete specific binding of the FFA2 orthosteric antagonist [^3^H]GLPG0974 (25) and acted as a PAM of the functional potency of the SCFA C3 in cAMP assays. Each of these is incompatible with AZ1729 acting as an orthosteric ligand. In recent times, applying modifications of the “operational model” of ligand function has allowed assessment of the binding affinity of allosteric ligands to GPCRs and the degree of cooperativity produced upon co-binding of an allosteric and orthosteric ligand (26). Although AZ1729 was unable to compete directly with [^3^H]GLPG0974 for binding to FFA2, it did produce a marked alteration in the observed affinity of C3 to do so. Analysis of this effect using the ACTM generated p*K_B_* = 6.84 ± 0.11 for AZ1729, and a very similar value for binding affinity of this ligand was obtained via analysis of the effect of AZ1729 on C3-mediated inhibition of forskolin-stimulated cAMP levels.

As initial studies on the activity of AZ1729 focused on assays that were PTX-sensitive and, therefore, mediated via G_i_-family G proteins, we were surprised that in the G_q_/G_11_-dependent IP_1_ assay AZ1729 was inactive as an agonist. Therefore, we turned to assessment of ERK1/2 phosphorylation. In Flp-In^TM^ T-REx^TM^ 293 cells induced to express hFFA2, C3 produced a large, concentration-dependent increase in phosphorylation of these proteins, as reported previously (15, 23). This clearly reflected an integration of G_i_ and G_q_/G_11_-initiated signals because a combination of pre-treatment with PTX and the G_q_/G_11_ inhibitor FR900359 was required to eliminate the effect of C3. By contrast, AZ1729 displayed very poor efficacy in this assay. Moreover, the modest effect of AZ1729 was unaffected by FR900359, indicating that it was not transduced via G_q_/G_11_, but blocked by PTX treatment. However, AZ1729 still binds to hFFA2 in this context and, indeed, acted rather as an insurmountable/allosteric antagonist of the function of C3 in the G_q_/G_11_-mediated IP_1_ assay.

Hence, our studies demonstrate that AZ1729 is an allosteric modulator that binds to a topographically distinct site from that of the endogenous orthosteric ligand (C3) in a manner that selectively enhances the coupling of FFA2 to G_i_-signaling pathways through a mechanism that primarily involves an increase in the efficacy of the orthosteric ligand (*i.e.* β value) with only a modest contribution made through an increase in the affinity of the orthosteric ligand (*i.e.* α value). Remarkably, the PAM activity of AZ1729 is selective to G_i_ signaling because AZ1729 acts in the opposite direction in the context of G_q/11_ signaling, namely decreasing the coupling efficacy of the orthosteric ligand to this signaling pathway. The selective nature of the PAM activity of AZ1729 is not only useful to probe the physiologically relevant signaling pathways downstream of FFA2 activation (as described below) but also establishes an important general principle that it is possible to generate PAMs to GPCRs that selectively enhance receptor coupling to one signaling pathway over another through a mechanism that is largely independent of the modulation of orthosteric agonist affinity but rather centered on the modulation of agonist efficacy.

Although the capacity of FFA2 to couple to various G proteins has been catalogued extensively in cell systems expressing the receptor in a heterologous manner, there remains a lack of information regarding FFA2-mediated signal transduction in cells endogenously expressing this receptor (7) and the contribution of different G proteins to initiation of these signals. It has been suggested that biased ligands, by preferentially activating specific receptor signaling pathways, can be valuable tools to assess the mechanistic basis of biological processes (39). Our experiments highlight this capability of biased ligands and give further information regarding the molecular mechanisms underpinning FFA2-mediated physiology by exploiting the unique pharmacology of AZ1729. In this regard, we assessed actions of AZ1729 in primary cells expressing FFA2 endogenously. The selective G_i_-mediated agonist effects of AZ1729 allowed prediction that because neutrophil chemotaxis toward SCFAs is a PTX-sensitive FFA2 effect (34, 36), then AZ1729 should be functional in such assays. Indeed it was, although much less efficacious than a maximally effective concentration of the well characterized neutrophil chemotactic agent fMLP, AZ1729 was as effective as C3 and functioned in a concentration-dependent manner. Moreover, it also acted as a PAM of the potency of C3. A concentration of AZ1729 that was ineffective in isolation enhanced the effect of a submaximal concentration of C3.

The molecular basis of FFA2-mediated anti-lipolytic effects was also assessed in murine adipocytes. As anticipated from previous work, following *ex vivo* “differentiation” of adipocytes produced from mouse epididymal fat, a process that acted to substantially up-regulate levels of FFA2 mRNA, AZ1729 was as effective, and much more potent, than C3 in inhibiting isoproterenol-stimulated lipolysis and release of glycerol (30–31). These data confirm the ability of FFA2 to reduce lipolysis in a G_i_-dependent manner, further demonstrating the utility of AZ1729 to identify FFA2-mediated effects that rely on G_i_-mediated signaling in physiologically relevant cells expressing the receptor endogenously.

In contrast to this was the lack of capacity of AZ1729 to modulate incretin secretion. C3 is able to stimulate an incretin effect in mouse colonic tissue and cultures (50, 51), promoting secretion of significantly enhanced levels of GLP-1 as confirmed herein. This was clearly a G_q_/G_11_-mediated effect as it was blocked by treatment with FR900359 and not reduced by treatment of the cells with PTX. Moreover, as anticipated for a defined G_q_/G_11_-mediated end point, AZ1729 was unable to recapitulate the incretin effect of C3, further confirming that FFA2 modulates GLP-1 secretion via a G_q/11_ mechanism in mouse colonic crypts. It is interesting to note in this regard that compound BTI-A-404, recently described as a selective and potent competitive inverse agonist of human FFA2/GPR43 (52), has been reported to promote GLP-1 secretion in a human cell line. Whether there is divergence between mouse and human FFA2 signal transduction at this end point thus requires further investigation. Together, these data highlight the ability of FFA2 to engage with different signaling pathways in physiological contexts as well as in transfected cell systems and shows that FFA2 pharmacology is tissue dependent. Thus, AZ1729 could be helpful in unraveling the signaling pathways behind the physiological roles of FFA2 and, potentially, its contribution to pathological states.

Overall, we provide detailed characterization of a novel FFA2 receptor allosteric ligand, AZ1729, which shows the unique property of being biased to generate functional effects only at PTX-sensitive effects of FFA2. In conjunction with the use of selective G protein pathway inhibitors this ligand can be used to specify G protein selection and will provide novel insights to the potential value of bias in G protein signaling in areas of FFA2 biology of potential clinical relevance.

## Materials and Methods

### Chemistry

All solvents and reagents were purchased from commercial suppliers and used without further purification. Analytical HPLC/MS was conducted on a Waters Zevo Q-TOF or Waters LCT Premiere mass spectrometer using an Acquity PDA (Waters) UV detector monitoring either at (*a*) 210 nm with an Acquity BEH C18 column (2.1 × 100 mm, 1.7 μm, 0.7 ml/min flow rate), using a gradient of 2% (v/v) acetonitrile in H_2_O (ammonium carbonate buffer pH 10) to 98% (v/v) acetonitrile in H_2_O or (*b*) 230 nm with an Acquity HSS C18 column (2.1 × 100 mm, 1.8 μm, 0.7 ml/min flow rate), using a gradient of 2% (v/v) acetonitrile in H_2_O (ammonium formate buffer pH 3) to 98% (v/v) acetonitrile in H_2_O. Preparative HPLC was conducted using a Waters Fraction Lynx Purification System using either (i) Xbridge Prep C18 5-μm OBD 19 × 150-mm columns. The mobile phase used was varying gradients of acetonitrile and 0.1 m HCO_2_H buffer; flow rate at 30 ml/min. ^1^H NMR spectra were generated on a Varian 300 MHz, Varian 400 MHz, Varian 500 MHz, or Varian 600 MHz instrument. Chemical shifts (δ) are given in parts per million (ppm), with the residual solvent signal used as a reference. NMR abbreviations are used as follows: br = broad, s = singlet, d = doublet, t = triplet, q = quartet, m = multiplet.

#### 

##### Synthesis of 1-[5-(3-Aminophenyl)-4-methyl-1,3-thiazol-2-yl]guanidine

1-[5-(3-Aminophenyl)-4-methyl-1,3-thiazol-2-yl]guanidine was synthesized according to the procedure described in PCT Int. Application WO 2007120096 A1 20071025 (53).

##### Synthesis of N-[3-(2-Carbamimidamido-4-methyl-1,3-thiazol-5-yl)phenyl]-4-fluorobenzamide

1-[5-(3-Aminophenyl)-4-methyl-1,3-thiazol-2-yl]guanidine (64 mg, 0.26 mmol) was dissolved in tetrahydrofuran (1.5 ml) and triethylamine (0.036 ml, 0.26 mmol) was added followed by 4-fluorobenzoyl chloride (41.0 mg, 0.26 mmol). The solution was stirred at room temperature for 3 h. The crude was concentrated and dissolved in Me_2_SO, filtered, and purified by HPLC, using a gradient of 5–95% acetonitrile in 0.1 m HCO_2_H as mobile phase, to yield 62 mg (64.9%) of *N*-[3-(2-carbamimidamido-4-methyl-1,3-thiazol-5-yl)phenyl]-4-fluorobenzamide. LC-MS *m/z* (ES+): 370.1 ^1^H NMR (Me_2_SO-*d*_6_): δ 2.31 (s, 3H), 6.88 (br s, 3H), 7.11 (d, 1H), 7.32–7.42 (m, 3H), 7.69 (d, 1H), 7.79–7.83 (m, 1H), 7.97–8.08 (m, 2H), 10.32 (s, 1H).

##### Other Compounds

Compound 1 was synthesized as described previously (54). 4-CMTB was synthesized as described previously (18).

### Cell Culture

All transformed cell lines used in these experiments were derived from Flp-In^TM^ T-REx^TM^ 293 cells (20) and designed to express the desired receptor, either human or mouse FFA2, on demand following induction with the antibiotic doxycycline (14, 15). In all cases, the receptor construct expressed was fused in-frame to enhanced yellow fluorescent protein (eYFP) at its C-terminal. The cells were maintained in Dulbecco's modified Eagle's medium without sodium pyruvate, supplemented with 10% (v/v) fetal bovine serum and penicillin/streptomycin mixture (Sigma, Poole, Dorset, UK) in a humidified atmosphere at 37 °C containing 5% CO_2_. To induce receptor expression, cells were incubated overnight with 100 ng ml^−1^ of doxycycline. Pre-treatments with PTX and/or FR900359 (22) were performed by incubating cells overnight with 100 ng ml^−1^ of PTX and/or for 30 min with 100 nm FR900359.

### cAMP Assays

cAMP assays were performed using a homogeneous time-resolved fluorescence (HTRF®) cAMP dynamic kit (CisBio Bioassays; CisBio, Codolet, France). Cells were plated at 2000 cells/well in low-volume 384-well plates, and inhibition of 1 μm forskolin-stimulated cAMP production was assessed following a 30-min co-incubation with test compounds. Outputs were measured by using a PHERAstar FS plate reader (BMG Labtech, Aylesbury, UK).

### IP_1_ Accumulation Assays

Were performed using a HTRF® IP1 dynamic kit (CisBio Bioassays). Cells were plated at 7500 cells/well in low-volume 384-well plates and incubated for 2 h at 37 °C with test compounds. Reactions were stopped according to the manufacturer's instructions and the signal was measured using a PHERAstar FS plate reader.

### Extracellular Signal-regulated Kinase (ERK) 1/2 Phosphorylation Assays

Assays were performed using a HTRF® phospho-ERK1/2 kit (CisBio Bioassays). Briefly, cells were plated at 70,000 cells/well in 96-well plates and then allowed to adhere for 3–6 h. Doxycycline was then added (100 ng ml^−1^) to induce expression of the receptor of interest, and cells were maintained in culture overnight. Prior to the assay, cells were serum starved for 4–6 h. After this, cells were treated with test compounds and incubated for 5 min at 37 °C, lysis buffer was then added and cells were incubated on a shaker for 30 min. Phospho-ERK1/2 reagents were added according to the manufacturer's instructions and signal measured using a PHERAstar FS plate reader.

### Membrane Preparation

Membranes were generated from Flp-In^TM^ T-REx^TM^ 293 cells either non-treated or treated with doxycycline (100 ng ml^−1^) to induce expression of FFA2. Briefly, cells were removed from flasks by scraping and centrifuged at 3,000 rpm for 5 min at 4 °C. Pellets were resuspended in TE buffer (75 mm Tris-HCl, 5 mm EDTA, pH 7.4) containing a protease inhibitor mixture (Roche Applied Science, West Sussex, UK) and homogenized with a 5-ml hand-held homogenizer. This material was centrifuged at 1,500 rpm for 7 min at 4 °C and the supernatant was further centrifuged at 50,000 rpm for 45 min at 4 °C. The resulting pellet was resuspended in TE buffer and protein content was assessed using a BCA protein assay kit (Pierce, Fisher Scientific, Loughborough, UK).

### Radioligand Competition and Three-way Binding Assays

Assays were carried out with 7.5 nm [^3^H]GLPG0974 ([^3^H]4-[[1-(benzo[*b*]thiophene-3-carbonyl)-2-methylazetidine-2-carbonyl]-(3-chlorobenzyl)amino]butyric acid) (25), Tris binding buffer (50 mm Tris-HCl, 100 mm NaCl, 10 mm MgCl_2_, 1 mm EDTA, pH 7.4), and the indicated concentrations of test compounds in a total assay volume of 200 μl in glass tubes. Binding was initiated by the addition of membranes (5 μg of protein per well). All assays were performed at 25 °C for 2 h before termination by the addition of ice-cold phosphate-buffered saline (PBS) and vacuum filtration through GF/C glass filters using a 24-well Brandel cell harvester (Alpha Biotech, Glasgow, UK). Each reaction well was washed six times with 1.2-ml aliquots of Tris-binding buffer. The filters were allowed to dry for 2–3 h and then placed in 3 ml of Ultima Gold^TM^ XR (PerkinElmer Life Sciences, Beaconsfield, UK). Radioactivity was quantified by liquid scintillation spectrometry. Specific binding was defined as the difference between binding detected in the presence and absence of 10 μm unlabeled GLPG0974.

### [^35^S]GTPγS Incorporation Assay

Assays (21) were carried out with reactions containing 5 μg of cell membrane proteins preincubated for 15 min at 25 °C in assay buffer (50 mm Tris-HCl, pH 7.4, 10 mm MgCl_2_, 100 mm NaCl, 1 mm EDTA, 1 μm GDP, and 0.1% fatty acid-free bovine serum albumin) with the indicated concentrations of ligands in a total assay volume of 500 μl in glass tubes. Reactions were initiated by addition of [^35^S]GTPγS (50 nCi/tube), and terminated after 1 h incubation at 25 °C by rapid filtration through GF/C glass filters using a 24-well Brandel cell harvester (Alpha Biotech, Glasgow, UK). Unbound radioligand was removed from the filters by three washes with ice-cold wash buffer (50 mm Tris-HCl, pH 7.4, and 10 mm MgCl_2_), and [^35^S]GTPγS binding was determined by liquid scintillation spectrometry. Nonspecific binding was measured in the presence of 100 μm GTPγS.

### Data Analysis and Curve Fitting

All data presented represent mean ± S.E. of at least three independent experiments. Data analysis and curve fitting were carried out using the GraphPad Prism software package version 5.0b. Concentration-response data were fit to three-parameter sigmoidal concentration-response curves. Three-way binding experiments were fitted using the Extended Allosteric Ternary Complex operational model Equation 1 described previously (26),

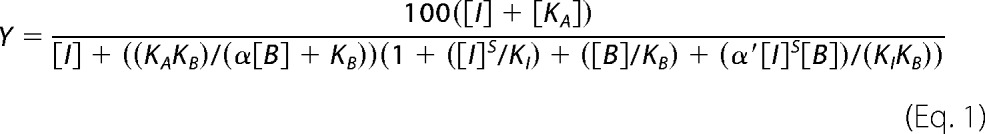
 where *Y* is the measured response, whereas *K_I_*, *K_A_*, and *K_B_* represent the equilibrium dissociation constants of the radioligand [^3^H]GLPG0974, unlabeled orthosteric ligand (C3 and compound 1), and allosteric modulator (AZ1729 and 4-CMTB), respectively, with [*I*], [*A*], and [*B*] indicating their concentrations. α is the measure of the affinity cooperativity factor between the allosteric modulator and the radioligand [^3^H]GLPG0974, whereas α′ is the measure of the affinity cooperativity factor between the allosteric modulator and unlabeled orthosteric ligand.

For functional studies (cAMP and IP_1_ accumulation assays), allosteric parameters were calculated by using the operational model Equation 2 as described previously (18),

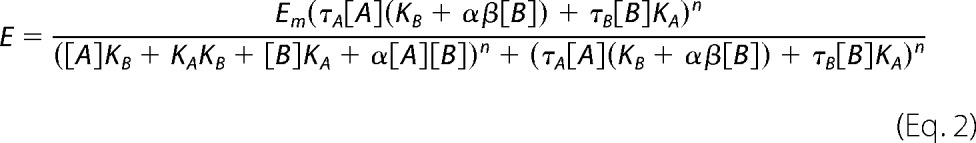
 where *E* is the measured response, and *A* and *B* represent the orthosteric and allosteric ligands, respectively. In this equation, *E_m_* is the maximal system response, α is a measure of the allosteric cooperativity on ligand-binding affinity, and β is an empirical measure of the allosteric effect on efficacy. *K_A_* and *K_B_* are measures of the binding affinities of the orthosteric and allosteric ligands, respectively. The value *n* represents the slope factor of the transduction function, whereas the abilities of the orthosteric and allosteric ligands to directly activate the receptor are incorporated through the values τ*_A_* and τ*_B_*. To fit experimental data to this equation, in all cases the system maximum (*E_m_*), slope (*n*), and *K_A_* functions were constrained, allowing for estimations of α, β, τ*_A_*, τ*_B_*, and *K_B_*. To fit the data presented in Figs. 5*D* and 7*C*, the α value was constrained to 1 to fit data for AZ1729 that did not perturb the potency of the allosteric ligand, whereas τ*_B_* was set to a value of effectively 0 to fit data for AZ1729 that did not produce any direct effect on its own.

### Animals

C57BL/6J mice purchased from Charles River (Margate, Kent, UK) were used to derive primary mouse adipocytes. FFAR2^−/−^ mice were generated by Deltagen (55) and backcrossed for 4 generations onto C57BL6/J at AstraZeneca, Mölndal, Sweden. Animals were cared for in accordance with national guidelines on animal experimentation.

### Derivation of Primary Mouse Adipocytes

Epididymal fat was collected from male mice, finely minced, and digested in HEPES buffer (100 mm HEPES, 120 mm NaCl, 4.8 mm KCl, 127 mm CaCl_2_, 4.5 mm glucose, 1.5% albumin, pH 7.4) containing 0.2% collagenase II (Sigma) for 30 min at 37 °C with rotation. After this time, digested material was filtered through a cell strainer (70 μm) and incubated for 15 min on ice. Sedimented stromal-vascular fraction was then collected and centrifuged at 1500 × *g* for 10 min. Cells were resuspended in DMEM (10% fetal bovine serum, 4 mm glutamine, 10 mm HEPES, 10 μg/ml of insulin, 25 μg/ml of sodium ascorbate) to 1000 cells/μl and seeded into 96-well plates, final volume was 150 μl. Cells were maintained at 37 °C, 5% CO_2_. Medium was replaced the day after cell isolation with DMEM containing 10 μm rosiglitazone. Medium was subsequently replaced every 2–3 days until the day of the experiment.

### Lipolysis Assay

Test compounds were prepared at the indicated concentrations in Hanks' balanced salt solution containing 25 mm glucose and 2% fatty acid-free bovine albumin serum. Differentiated primary mouse adipocytes were washed three times with Hanks' balanced salt solution prior to the addition of test compounds. Cells were then incubated at 37 °C for 2 h and, after this time, 50 μl of cell supernatants was transferred to a 96-well plate. Glycerol concentration in the supernatants was measured by the addition of 50 μl/well of free glycerol reagent (Sigma). Plates were then incubated at 37 °C for 5 min before absorbance at 540 nm was measured using a Pherastar FS microplate reader.

### Human Neutrophils

#### 

##### Isolation and Migration Assay

Neutrophils were isolated from human whole blood using the MACSxpress® Neutrophil Isolation Kit according to the manufacturer's protocol (Miltenyi Biotec, Bergisch Gladbach, Germany). Isolated neutrophils were resuspended in RPMI 1640 containing 0.5% fatty acid-free bovine serum albumin. Test compounds were prepared at the indicated concentrations in the same buffer and added at the bottom of a 24-well plate (Sigma). Inserts were then mounted to the plate and neutrophils were added (5000 cells/μl). Cells were incubated at 37 °C for 1.5 h and migrated cells were then collected and counted using a hemocytometer.

##### Flow Cytometry

Isolated human neutrophils were incubated for 30 min with CD15-FITC or isotype-FITC antibodies (Biolegend, London, UK). Cells were analyzed with a BD FACSCalibur^TM^ platform (BD Biosciences) and analyses were performed with FlowJo software (TreeStar, Eugene, OR).

### Quantitative PCR

Total RNA from mouse adipocytes was prepared by cell lysis and purification using miRNeasy micro kits (Qiagen). The miScript Reverse Transcription Kit (Qiagen) was used for cDNA preparation. TaqMan Gene Expression Assays (Applied Biosystems) were used for determination of gene expression. The expression of TATA-binding protein was used as control and data were analyzed using an ABI7900HT machine with SDS 2.2 software. Data are presented as RQ values (2^−ΔΔ^*^CT^*) with expression relative to endogenous control and control sample at day 1 of differentiation.

### Mouse Colonic Preparations and GLP-1 Release Assays

Murine colonic crypt cultures were prepared as previously described (56). Briefly, the muscle layer from the colon and rectum was removed. The remaining tissue was washed in PBS, chopped, and digested with 0.4 mg ml^−1^ of collagenase XI (Sigma) at 37 °C into cell clusters resembling crypts. Crypts were seeded onto 24-well plates coated with Matrigel (Corning), and incubated overnight at 37 °C and 5% CO_2_. Experiments were performed after 1 day *in vitro*, except for the PTX experiments, which were performed after 2 days. Crypts were incubated with 100 nm FR900359 for 30 min or 200 ng ml^−1^ of PTX for 16–18 h prior to addition of test ligands. Test ligands were added in 138 buffer (mm: HEPES 10, NaCl 138, KCl 4.5, NaHCO_3_ 4.2, NaH_2_PO_4_ 1.2, CaCl_2_ 2.6, MgCl_2_ 1.2) containing 500 kallikrein inhibitor units ml^−1^ of aprotinin, 10 μm amastatin, and 0.1 mm diprotin A to inhibit breakdown of GLP-1, and incubated for 2 h at 37 °C and 5% CO_2_. Supernatants were removed, and cells were lysed using lysis buffer (50 mm Tris-HCl, 150 mm NaCl, 0.5% C_24_H_39_NaO_4_·H_2_O, 1% Igepal CA-630) containing Complete EDTA-free protease inhibitor mixture. Secretion supernatants were centrifuged at 4 °C and 8,000 × *g*, and lysates at 4 °C and 21,130 × *g* to pellet cell debris. Active GLP-1 concentrations were determined by ELISA (Millipore). Secretion was expressed as GLP-1 released over the 2-h incubation period as a percentage of the total crypt GLP-1 content.

## Author Contributions

G. M., A. B. T., A. M. M., and N. D. developed and coordinated the project, D. B., C. E. M., I. D., and M. K.-S. performed the experiments, K. N. and A. U. P. discovered and synthesised AZ1729, E. S., G. M. K., and E. K. provided novel reagents. G. M. and D. B., with the assistance of others, wrote the manuscript.

## Footnotes

2 The abbreviations used are:

SCFA: short chain fatty acid
eYFP: enhanced yellow fluorescent protein
fMLP: *N*-formyl-methionyl-leucyl-phenylalanine
GLP-1: glucagon-like peptide-1
GPCR: G protein-coupled receptor
PAM: positive allosteric modulator
PTX: pertussis toxin
GTPγS: guanosine 5′-*O*-(thiotriphosphate)
IP_1_: inositol monophosphate
AZ1729: *N*-[3-(2-carbamimidamido-4-methyl-1,3-thiazol-5-yl)phenyl]-4-fluoro benzamide
4-CMTB: 4-chloro-α-(1-methylethyl)-*N*-2-thiazolyl-benzeneacetamide
compound 1: 3-benzyl-4-(cyclopropyl-(4-(2,5-dichlorophenyl)thiazol-2-yl)amino)-4-oxobutanoic acid
GLPG0974: (4-[[1-(benzo[*b*]thiophene-3-carbonyl)-2-methylazetidine-2-carbonyl]-(3-chlorobenzyl)amino]butyric acid).
